# A review on self-healing featured soft robotics

**DOI:** 10.3389/frobt.2023.1202584

**Published:** 2023-10-26

**Authors:** Md. Ariful Islam, Labanya Talukder, Md. Firoj Al, Subrata K. Sarker, S. M. Muyeen, Prangon Das, Md. Mehedi Hasan, Sajal K. Das, Md. Manirul Islam, Md. Robiul Islam, Sumaya Ishrat Moyeen, Faisal R. Badal, Md. Hafiz Ahamed, Sarafat Hussain Abhi

**Affiliations:** ^1^ Department of Mechatronics Engineering, Rajshahi University of Engineering and Technology, Rajshahi, Bangladesh; ^2^ Department of Electrical Engineering, Qatar University, Doha, Qatar

**Keywords:** self-healing, soft robotics, soft actuator, extrinsic, hydrogel, intrinsic, dielectric elastomer actuator, wearable robots

## Abstract

Soft robots are becoming more popular because they can solve issues stiff robots cannot. Soft component and system design have seen several innovations recently. Next-generation robot–human interactions will depend on soft robotics. Soft material technologies integrate safety at the material level, speeding its integration with biological systems. Soft robotic systems must be as resilient as biological systems in unexpected, uncontrolled situations. Self-healing materials, especially polymeric and elastomeric ones, are widely studied. Since most currently under-development soft robotic systems are composed of polymeric or elastomeric materials, this finding may provide immediate assistance to the community developing soft robots. Self-healing and damage-resilient systems are making their way into actuators, structures, and sensors, even if soft robotics remains in its infancy. In the future, self-repairing soft robotic systems composed of polymers might save both money and the environment. Over the last decade, academics and businesses have grown interested in soft robotics. Despite several literature evaluations of the soft robotics subject, there seems to be a lack of systematic research on its intellectual structure and development despite the rising number of articles. This article gives an in-depth overview of the existing knowledge base on damage resistance and self-healing materials’ fundamental structure and classifications. Current uses, problems with future implementation, and solutions to those problems are all included in this overview. Also discussed are potential applications and future directions for self-repairing soft robots.

## 1 Introduction

Robotics or robots are the science and engineering products or devices that can be multi-functional and multi-purpose versatile programmable systems. These robots might be soft or hard. In contrast to soft-bodied robots, rigid-bodied complex robots have low behavioral density and bio-inspiration with high precision, speed, and forced applications. Soft robots bend, stretch, and reverse (Laschi et al., 2016). Human skin can heal wounds of various sizes, suggesting it may restore mechanical and electrical functions. Wear and tear, corrosion, and damage will break manufactured electrical equipment. Durable soft electronic compositesmay benefit from self-healing chemistry.

**FIGURE 1:**
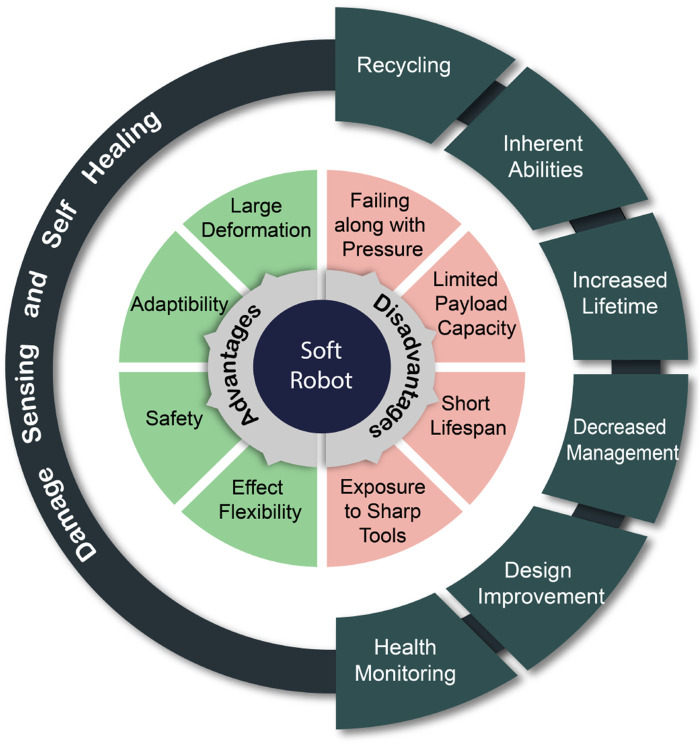
SH soft robotics.

Long-term usage in sensitive areas limits soft robots’ lifetime due to their susceptibility to harm. The robot’s adaptation to uncontrolled conditions is a significant issue. Systems should be able to recover from harm like biological ones. Materials with high healing properties allow the soft robot to heal its damaged parts and give them a long time of usability (Figure 1) (Yap et al., 2020). More flexible, adaptive, and obedient than rigid robots, soft robots are appropriate for many applications. Soft robotics, an emerging field, produces robots with more compliance, flexibility, and variety using malleable materials. Soft robots use polymers, elastomers, and hydrogels. Soft, durable, self-repairing textiles are challenging to create. Another issue is accurate soft robot control algorithms. These promising advancements provide soft robots with three dimensions. First, develop robotic materials with soft and solid components. This integration enables versatile soft robots with realistic self-healing, sensory awareness, and growth. Second, understand soft robot dynamics. Specialized calculations expand this insight. Finally, novel control and learning strategies improve soft robot performance (Yasa et al., 2023). Soft magnetic robots are promising but need major manufacturing, design, and functional development to be practical. Soft magnetic robot development involves complex materials selection, actuation, and functionalization. Design—including magnetic materials, geometry, and construction—determines the robot’s performance, efficiency, and adaptability. Recent breakthroughs allow bionics to construct soft robots. Two key aims are how bioinspired methods may enhance soft magnetic robot design and actuation for realistic motion (Miao and Sun, 2023). Using magnetically actuated actuators, magnetically responsive materials, and magnetically controlled shape memory alloys, untethered soft magnetic robots can use electromagnetic, magnetostrictive, and shape memory alloy actuation. Multiple design strategies, actuation methods, and functionalizations may construct life-like soft magnetic robots for locomotion, manipulation, and sensing. Soft magnetic robots for swimming, crawling, gripping, manipulating, and detecting the surroundings (Miao and Sun, 2023).

**FIGURE 2:**
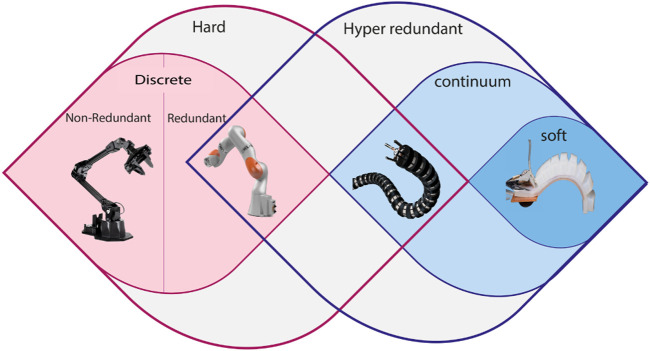
Materials and degrees of freedom-based robot categorization. Empty sets are indicated by hatching.

Soft actuators for small-scale systems focus on development methods for nanoscale to centimeter-scale robotic applications. Soft actuators use flexible materials for nanoscale to centimeter-scale robotic applications. These materials are usually polymers, elastomers, or hydrogels. Soft actuators have several advantages over rigid actuators. These include energy efficiency, conformity, and low weight. This research designs and builds lightweight, compliant, and energy-efficient soft actuators for small-scale robots. Small-scale robots require actuators for quick, precise movement in tight spaces (Hines et al., 2017). Soft synthetics deteriorate quickly through wear, cracking, and environmental degradation. Harm resistance protects against unforeseen shocks. Soft robotics could be damage-resistant when interacting with biological systems in unknown settings (Bilodeau and Kramer, 2017). Damage resilience is performance after injury. The capacity of robots to self-repair, like biological systems, would further distinguish soft robots from rigid-bodied ones. Soft, self-healing robots provide skill, safety, flexibility, a broad range of motion, and human contact (Terryn et al., 2021). Soft robots can navigate confined areas and challenging environments, unlike sophisticated robots whose materials limit them. Complex rigid-frame robots need precise control of individual links and joint actuators. They are suitable for mass production but stay the same. Most industrial robots serially connect complex parts like human limbs (Saigo et al., 2019). Robots may become outdated under certain conditions. Since redundant robots have limitless postures from the same final effector motion, inverse kinematics is challenging to fix. Too many degrees of freedom in kinematically redundant manipulators (Chiaverini et al., 2016). Hyper-redundant robots have multiple joints and redundant components. Due to their joint-less, continuous body, hyper-redundant Continuum Robots have a completely different mechanical structure. With its limitless degrees of freedom, continuum and soft robotics research redundant or flexible mechanisms and their applications to autonomous systems (Rao et al., 2021). Figure 2 shows the clear categorization, and Table 1 shows the complete characteristics comparison of all these sets of robots.

**TABLE 1:** Characteristics comparison of hard robotics and soft material robotics.

		Rigid body	Discrete hyperredundant	Hard continuum	Soft
	Degree of Freedom	Few	Large	Infinite	Infinite
	Actuators	Few, discrete	Many, Discrete	Continuous	Continuous
Attributes	Material	Metal, Plastics	Metal, Plastics	Shape memory alloy	Rubber, electroactive polymer
	Simulation	Easier	Tough	Complicated	Complicated
	Material Strain	None	None	Small	Large
	Accuracy	Very high	High	High	Low
	Dexterity	Low	High	High	Low
	Safety	Dangerous	Dangerous	Dangerous	Safe
Capabilities	Load Capacity	High	Lower	Lower	Lowest
	Manipulable Objects	Fixed sized	Variable size	Variable size	Variable size
	Conformability to Obstacles	None	Good	Fair	Highest
	Working Environment	Structured only	Structured and unstructured	Structured and unstructured	Structured and unstructured
	Controlling ability	Easier	Medium	Complicated	Complicated
Design	Position sensing	Easier	Tough	Complicated	Complicated
	Path planning	Easier	Tough	Complicated	Complicated

“Soft roboticists” examine nature’s soft, flexible, and adaptable bodies to design robots with capabilities beyond rigid robots. As their equipment and electronics become smaller, lighter, and less stiff, they blend better with their environment and people (Terryn et al., 2021). Ceramics, metals, polymers, and composites are the most prevalent healing materials. Flexible sensor interfaces must be biocompatible and have the same mechanical characteristics as target tissues to decrease mechanical mismatch and immune reactivity (Roels et al., 2020).

## 2 SH soft robotics structure

**FIGURE 3:**
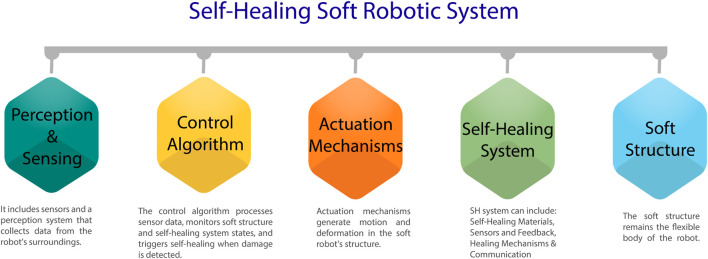
Structured flow of Self-Healing Soft Robotic System.

Soft robots need structure to function and adjust freely. To maximize soft robot flexibility and usage, use touchable materials. Self-healing passive structures, the material frameworks that hold actuators and sensors together, are a simple approach to improve them. Soft robotics researchers want materials with variable stiffness, and soft robotic systems might adapt to new situations and create force using this. The structure of a self-healing soft robotic system is illustrated in Figure 3. Table 2 shows the characteristics comparison of broadly used actuators, and the remaining few are also described briefly.

**TABLE 2:** Characteristics comparison of soft actuators, liquid crystal elastomers, hydrogels, inflatable actuators, shape memory materials.

	Soft actuators	Liquid crystal elastomers	Hydrogels	Inflatable actuators	Shape memory materials
Definition	Components enable movement or actuation through compliant and flexible materials	Soft materials exhibiting large shape changes in response to external stimuli	Soft materials composed of hydrophilic polymers that can absorb and retain large amounts of water	Actuators using flammable materials to induce movement or change in the shape of a soft structure	Materials that undergo reversible shape changes in response to external stimuli
Characteristics	Compliant, flexible; diverse material options	Large shape changes, anisotropic mechanical properties	High water content, responsive to environmental conditions	Use of flammable materials for actuation	Retain multiple stable shapes and transition between them
Strengths	High compliance, adaptability, safe interaction	Large shape changes, precise control, tunable mechanical properties	Biocompatibility, responsiveness, mimic biological tissues	Unique actuation mechanism, lightweight design	Reversible shape changes, responsiveness to stimuli
Weaknesses	Limited force output, the potential for energy inefficiency	Complexity in fabrication, potential material limitations	Limited mechanical strength, can be sensitive to environmental conditions	Safety concerns due to the use of flammable materials	Limited control over shape transition and sensitivity to stimuli
Applications	Gripping, manipulation, locomotion, prosthetics, biomedical devices	Soft robots, artificial muscles, adaptive optics	Soft sensors, drug delivery systems, biomedical devices	Soft robotics, lightweight applications	Morphing structures, reconfigurable devices, smart materials
Examples	Pneumatic/hydraulic actuators, electroactive polymers	Liquid crystal elastomer composites, light-responsive polymers	pH-responsive hydrogels, drug delivery systems	Combustible gas actuators	Shape memory alloys, shape memory polymers

### 2.1 Inherent healing polymers

Polymers with inherent healing may fix damage without additional help. Dynamic chemical linkages like reversible covalent bonds or supramolecular interactions may break and rejoin to cure these materials. Internal healing processes such as Diels–Alder reactions, reversible hydrogen bonding, and physical interactions are covered in the study. These materials can be repaired repeatedly, making them ideal for long-term use. Polymers with intrinsic healing characteristics have promising prospects for enhancing the durability and dependability of various goods and constructions (Irzhak et al., 2022).

### 2.2 Microcapsules

Microcapsules containing healing agents are embedded in the polymer matrix to create capsule-based self-healing systems. When damaged, capsules release healing agents. The healing agent may repair fractures after interacting with other materials. This approach works effectively for chemical-treatable, non-self-healing materials (Blaiszik et al., 2009). Microcapsules have shells around their cores. Polymers or other materials make up the shell. The core contains the curative ingredient, which may be resin, adhesive, or polymer precursor. After an injury, the microcapsules break, releasing the healing agent. The therapeutic component then interacts with the surrounding environment, frequently chemically, to fill and heal the fissure or defect, restoring structural integrity (Calvino and Weder, 2018).

### 2.3 Microvascular networks

Microvascular networks are a more complex self-healing strategy consisting of a distributed network of microscopic channels or capillaries. When damaged, these channels break, releasing a healing chemical to mend the substance. Microvascular networks distribute healing chemicals more evenly across a broader region than capsule-based systems (Toohey et al., 2007). The microvascular network responds autonomously to material degradation like cracks or fractures. After identifying damage, controlled valves or capillary action release a curative material from microvessel reservoirs or chambers into the damaged area. The healing agent penetrates and closes cracks, restoring structural integrity (Gonai et al., 2020).

### 2.4 Soft actuators

Soft Actuators are available in various forms and dimensions and can transform mechanical energy into motion. The ability of soft actuators to generate motion in robots has attracted much interest and is essential to the development of soft robotics (Bilodeau and Kramer, 2017). Electricity, magnetic fields, thermal energy, pressurized fluids, and chemically induced mass transfer may cause kinematic motion. Soft actuators may strain flexible materials. This section discusses current damage-resilient actuator research (Wang et al., 2019). Due to their adaptability and endurance, soft robots use soft actuators as transducers. Hydroelectric self-repairing electrostatic actuators use hydraulic and electrostatic forces. Many substances trigger self-healing. Poly Multi-User MEMS, Silicon-On-Insulator, and Metal MUMPs are standard actuator production methods. Soft robots need actuation materials to manipulate and detect muscles (Kumar et al., 2021). For micro- and nano-scale robotics, soft actuation needs expensive or complex external equipment despite its many advantages. Although each solution serves a different purpose, multiple ways have emerged as the power source for any unconnected soft robotic system has become the norm. Each strategy needs to combine several tactics effectively. Performance must include rapid response, reliability, actuation, controllability, and portability (Tang et al., 2021).

Dielectric Elastomer Actuators (DEAs) provide a potential actuation method. Due to their high actuation strain, broad frequency range, and energy density, dielectric elastomers are suitable actuators. By charging the electrodes and squeezing the elastomer-based dielectric layer between them, DEAs can reduce actuator thickness and expand the actuator area (Kumar et al., 2021). Silicones, acrylic elastomers, and other pliable nonconductive materials are suitable for use as DEA dielectrics. DEAs may be either semi-liquid or solid. The dielectric is thin, but it sometimes takes extremely high electric potentials to produce significant shape change (Bilodeau and Kramer, 2017; Zhang et al., 2019).

Hydrogels are polymeric matrices with hydrogel water and hydrophilic polymer chains. Hydrogels combine water into large structures. Hydrogels expand because the polymer matrix attracts and absorbs water molecules into the interstitial spaces between the elongated polymer chains. In soft robots, diffusion-limited actuation is commonplace. Materials scientists now research self-healing hydrogels separately. Since self-repairing hydrogels have yet to reach soft robotics, this section focuses on the future utilizing existing technology (Tan et al., 2021). Developing self-healing hydrogels that are more durable, efficient, and versatile is essential for various applications, including medical devices, sensors, and actuators. Microcapsule-based self-healing hydrogels include healing ingredients. Hydrogel damage breaks microcapsules, releasing healing chemicals. Healing chemicals spread across the hydrogel network to repair injury (Liu et al., 2019). In soft robotics, this diffusion-limited process is a standard actuation method. New studies establish self-healing hydrogels as an independent study area for materials scientists. This section focuses mainly on the future by using technology already on the market because hydrogels that can mend themselves have yet to reach the soft robotics community (Lee et al., 2020b; Su et al., 2021;). Create self-healing hydrogels by creating materials that can mend themselves after damage. Use reversible bonds to break and rebuild. There are several types of self-healing hydrogels with different mechanisms. Self-healing hydrogels mend quickly. Materials’ quick self-repair is essential in medical equipment and wearable robotics. Robust, durable, self-healing hydrogels are another critical project. Many applications, especially those with extensive usage and extreme environments, such as industrial or environmental settings, need materials to be durable. Self-healing hydrogels may alter medicinal, industrial, and construction industries. Self-healing hydrogels should be fast, effective, strong, and durable (Taylor, 2016). Photoresponsive hydrogel-based soft robots test the potential of photoresponsive hydrogels for novel soft robot functions. This hydrogel changes with light. Using photoresponsive hydrogels in soft robotics offers benefits such as light-based control. Photoresponsive hydrogels are easy to make and can be used to control the movement and behavior of soft robots with high precision and efficiency, develop new functions and capabilities, and control their movement and behavior (Liu et al., 2021).

Liquid Crystal Elastomers (LCEs) comprise crystal molecules called mesogens connected to a bendable polymer backbone. Mesogens may rearrange and limit molecular movement due to their flexibility. Heat or electricity reorient mesogens. Mesogen reorientation causes backbone strains and LCE bulk strains. Heat dispersion delays thermally activated LCEs during the 10-s relaxation period. So, they require active cooling. The average molecular shape changes from spherical to spheroidal when the polymer transitions from nematic to isotropic during actuation. This alteration matches the phase shift. Activated ferroelectric liquid crystals have mesogens with inherent polarization. Mesogens realign in an electric field, creating bulk stresses and strains. Ferroelectric elastomers need less applied field than polymers and dielectrics. Reduce the applied voltage to match material thickness (O’Halloran et al., 2008; Ula et al., 2018).

Sensors made of an intrinsic self-healing and stretchable cross-linked network are designed to be compatible with soft robots, and they can be used to detect a variety of stimuli, including pressure, temperature, and chemicals. Soft robots require sensors to function under challenging conditions and survive harm. A cross-linked polymer chain network makes up the sensor. Stretchable and self-healing, this network is ideal for soft robotics. The sensor measures polymer network resistance to detect various inputs. Sensor stimulation alters network resistance. Measure this resistance change to determine stimulus kind and magnitude. Self-healing and flexible sensors make soft robots more durable, trustworthy, and multifunctional (Dai et al., 2023).

### 2.5 Smart morphing materials (SMMs)

Smart materials like SMMs alter morphologically in response to inputs. Due to their versatility, these materials are famous for research. The appropriate inputs may distort, sustain, and reverse specific polymers. This SME manufactures biopolymers, robotics, and aeronautical engineering polymers. Also studied are heat-shrinkable packaging films, intelligent fabrics, actuators, and self-expanding stents for vascular and other tissue repair. Soft-robot programming is possible. These robots use shape-shifting flexible polymers, which encode shape-shifting and shape memory soft materials, which can program complex form changes on demand but do not encode shape-shifting (Scalet, 2020). Metal SMAs and polymer SMPs fall under the shape memory materials category (SMPs). It may undergo plastic deformation from a preset state under controlled heating and revert to that condition. Soft robotics applications use SMAs and SMPs because of their shape-recovering abilities (Huang et al., 2019).

### 2.6 Nanomaterial-based healing

Nanomaterial-based healing entails embedding nanoparticles, such as nanocapsules or nanotubes, into a host material, allowing it to self-repair when damaged. Upon the occurrence of damage, the activation of these nanoparticles ensues, initiating a process for repair. For example, nanocapsules can release therapeutic substances upon rupture, while nanotubes can enhance structural integrity. Using nanomaterials in healing processes has many advantages, including enhanced product durability, decreased expenses related to maintenance, and heightened safety measures. Moreover, this approach aligns with sustainability goals by limiting waste generation. The challenges included in this context are the exact manipulation of nanoparticle dispersion, the capacity to scale up the process, and the assurance of compatibility between nanoparticles and the host materials (Kushwaha et al., 2022).

### 2.7 Modular design

One approach to fabricating elastomers is by a vat photopolymerization technique. The proposed system involves a combined thiol/acrylate chain/step-growth polymerization mechanism, which exhibits the ability to undergo self-healing over numerous cycles of injury and subsequent healing. The thiol/acrylate mixed chain/step-growth polymerization system is combined. Subsequently, the amalgamation is subjected to a vat photopolymerization technique for 3D printing. Subsequently, the printed elastomer undergoes a curing process by exposure to UV radiation. The elastomer has achieved self-healing capabilities, enabling convenient repair by reuniting the two fractured ends. Implementing robots with many linked components enables the isolation and repair of a damaged component without causing any disruption to the other components of the robot (Gomez et al., 2021).

## 3 Classification of SH Polymer

**FIGURE 4:**
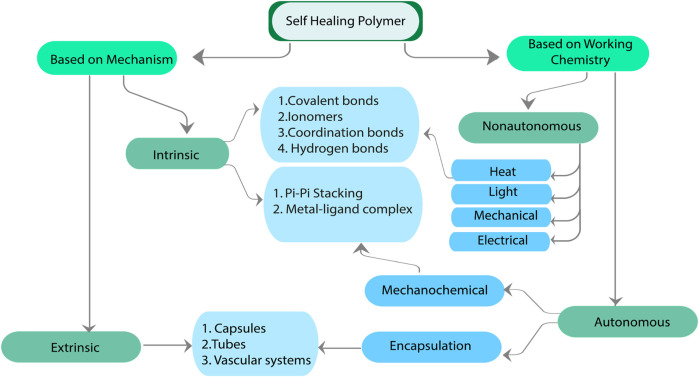
Classification of SH polymer.

Self-healing (SH) polymers can automatically repair themselves in response to injury without requiring manual intervention for detection or repair. According to their underlying mechanisms and working chemistry, self-healing polymers can be categorized into two groups, as illustrated in Figure 4 (Terryn et al., 2021; Goyal et al., 2022).

Based on working chemistry, SH polymers can be classified as Autonomous and Non-autonomous. In non-autonomous systems, the healing mechanism needs to be initiated by an external trigger. Heat, light, mechanical or chemical, or any combination of these may be the stimuli (Priyadarsini et al., 2021). Non-autonomous materials may mend themselves when exposed to heat or ultraviolet light. In Autonomous systems, polymers can undergo self-healing processes without requiring external triggers. They can sense the damage and initiate the repair process on their own.

In an alternative perspective, self-healing methods can be categorized into two distinct classifications: extrinsic and intrinsic. Autonomous self-healing polymers combine intrinsic and extrinsic mechanisms to provide a comprehensive healing response. Two types of intrinsic healing can be distinguished by their underlying molecular principle: supramolecular interactions and reversible covalent bonds. Extrinsic healing systems require external healing material in the capsule or the vascular network. Table 3 shows the complete characteristics comparison of Intrinsic, Extrinsic, Autonomous, and Non-Autonomous Materials based soft robots.

**TABLE 3:** Characteristics comparison of intrinsic, extrinsic, autonomous and non-autonomous materials.

	Intrinsic materials	Extrinsic materials	Autonomous materials	Non-autonomous materials
Definition	Materials with inherent softness, compliance, and desired properties for soft robotics applications	Conventional materials modified or combined with other materials to exhibit soft robotic properties	Materials can sense and respond to environmental stimuli without external control or power sources	Materials requiring external control or power sources to achieve desired functionalities or responses
Properties	Soft, compliant, flexible, inherent properties	Combination of soft and rigid materials, tailored properties for softness	Sensing and actuation capabilities, self-responsive behavior	Reliance on external control systems or power sources for functionality
Strengths	Inherent properties, simplicity, suitable for compliant applications	Versatile combinations, hybrid properties, broad material options	Self-adaptation, responsiveness, reduced need for external control	Precise control, programmability, specific functionality
Limitations	Weaknesses and limited material options may lack rigidity or specific functionalities	Complexity in fabrication, potential stiffness limitations	Sensing and actuation may be limited to specific stimuli or environmental conditions	Dependence on external control systems increased complexity
Examples	Elastomers, polymers, and gels	Hybrid structures with modified rigid materials	Shape memory alloys, pH-responsive hydrogels	Pneumatic actuators, electrical motors, programmable controllers
Applications	Soft grippers, wearable devices	Robotic exoskeletons, prosthetic limbs	Self-adaptive structures, responsive sensors	Controlled actuation, programmable behavior

### 3.1 Intrinsic self-healing

**FIGURE 5:**
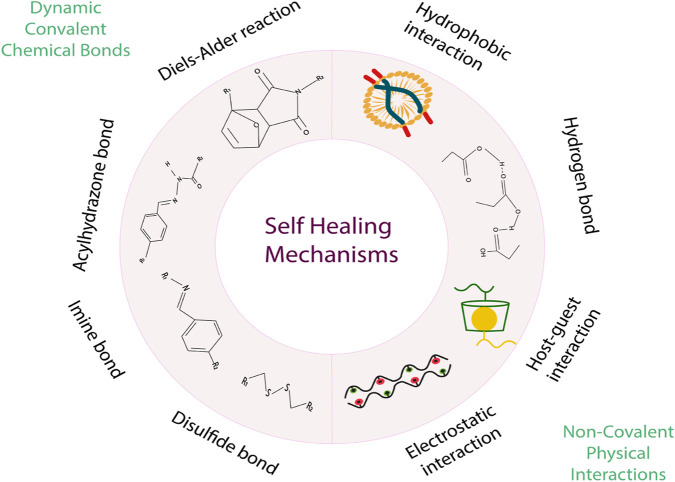
A summary of Dynamic covalent chemical bonds and Non-covalent physical interactions used in the self-healing polymer mechanism.

Intrinsic self-healing requires extrinsic cues to break down previously established connections before forming new ones. As a result, it is sometimes referred to as stimulus-responsive self-healing (Khan et al., 2018). Categorizing intrinsic self-healing systems according to their underlying healing mechanism is also possible. The two most common connection forms are chemical bonding and physical connection (Figure 5). The involvement of non-covalent bonds, like diffusion, entanglement,*π*-*π* -stacking, ionic and hydrophobic contacts, hydrogen bonding, and metal coordination plays a vital role in physical self-healing. Intrinsically polymers feature a variety of dynamic covalent links, such as acyl hydrazone bonds, Diels–Alder reactions, boronate ester bonds, disulfide exchanges, imine bonds, alkoxyamine bonds, diselenide bonds, silyl ether linkages, and hindered urea connections, amongst others. For polymers to repair themselves spontaneously, they need to undergo a series of dynamic processes on many length scales. Polymer chains and molecular interactions should be flexible in the final product. Fragmented interactions must be physically near the macroscopic level for dynamic rearrangement (Xu et al., 2020).

#### 3.1.1 Diels–Alder cycloaddition reaction

The Diels–Alder cycloaddition reaction is a chemical reaction that can be used to design self-healing materials. Diels and Alder are credited with the discovery of the Diels–Alder reaction. A conjugated Diene and a substituted Alkene undergo [4 + 2] cycloaddition to form substituted cyclohexene. This reaction is a [4*π*S+2*π*S] cyclo-addition between a 4*π*-electron (the Diene composition) and a 2*π*-electron (the Aldol composition) system, regulated by orbital symmetry (Terryn et al., 2022). Temperature can be used to regulate the rate of the DA reaction because it is thermally reversible. Because this method does not necessitate high temperatures, it is ideal for polymers (Ratwani et al., 2022).

The first example of self-healing materials based on the Diels–Alder reaction was reported in 2001 by White and Sottos, who demonstrated the self-healing behavior of a thermosetting epoxy polymer containing furan and maleimide functional groups.

#### 3.1.2 *π*-*π* stacking

*π*-*π* interactions are a type of supramolecular interaction that involves overlapping *π*-orbitals in aromatic molecules. In self-healing materials,*π*-*π* interactions can create dynamic and reversible bonds that allow the material to repair itself when damaged (Stukalin et al., 2013). In a self-healing polymer, *π*-*π* interactions can be designed between the polymer chains, forming a network of aromatic rings. When the material is damaged, the *π*-*π* interactions are disrupted. Still, when the material is heated or exposed to the appropriate stimuli, the aromatic rings can realign, and the *π*-*π* interactions can reform, allowing the material to repair itself.

#### 3.1.3 Ionomeric self-healing system

Thermoplastic ionic copolymers (ionomers) made of hydrocarbon chains with carboxylic acid groups and metal or quaternary ammonium ions are used. Example: EMAA. EMAA’s ionic segments can form clusters broken and reconstituted by temperature and UV radiation.

### 3.2 Extrinsic self-healing

Extrinsic self-healing uses an external healing material, encapsulated or vascularized. The micro-capsule or hollow fiber used to inject the vessels into the polymeric matrix is an example of an extrinsic healing approach.

#### 3.2.1 Capsule-based self-healing

**FIGURE 6:**
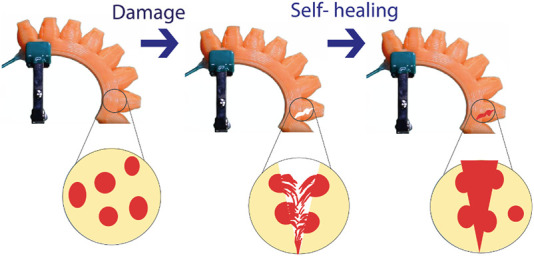
Self-healing by Encapsulation mechanism.

In the micro-encapsulation method, cracks cause a release of a low-viscosity healing agent enclosed in the capsules, which then fills and repairs the cracks. Additionally, capsules can be either spherical or cylindrical; they can hold 83%–92% of a liquid therapeutic ingredient and are made from urea-formaldehyde polymers (Van Tittelboom and De Belie, 2013; Mobaraki et al., 2020). The encapsulated method has the disadvantage that the therapeutic substance within the capsule can only be accessed after the capsule has been opened (Figure 6).

White et al. (2001) proposed this polymer matrix self-healing method. It presented a healing system for the ring-opening metathesis (ROMP) of dicyclopentadiene using Grubbs’ catalyst. It is an abbreviation for ring-opening metathesis (DCPD). Adding Grubbs’ catalyst to an epoxy matrix that already included microcapsules constructed of urea-formaldehyde (UF) shells filled with DCPD resulted in a considerable increase in the toughness of the epoxy.

By encapsulating epoxy ester resin inside poly (urea-formaldehyde-melamine) microcapsules, Cotting et al. (2019) created an epoxy coating that can repair itself if damaged. An epoxy coating system consisting of three layers was given the addition of the filled microcapsules. When stressed by a mechanical flaw, the coating system with microcapsules had a considerable self-healing effect, and the greater concentration (15 wt%) offered superior self-healing protection and anti-corrosive performance.

#### 3.2.2 Vascular self-healing

Chemicals and catalysts activate the vascular self-healing mechanism in capillaries and other hollow channels. It starts a chain reaction of repair activities. When microcracking releases the healing ingredient from the arteries or hollow canals, it travels through a network of nano- or micro-sized vascular tubes (Nik Md Noordin Kahar et al., 2021). Breaking capillaries releases the healing agent, which repairs microcracks. Vascular networks can be 1-, 2-, or 3-dimensional, and the integrated vascular network allows 2D and 3D structures to heal repeatedly in the exact location. In addition to containing healing agents, vascular networks reinforce polymeric matrices (do Nascimento, 2021).

## 4 Application OF SH soft robotics

Unlike traditional robots, which are limited to using rigid connections, soft robots may squeeze, stretch, climb, and expand in addition to performing standard robot operations like grabbing and movement. For example, a rigid link-based approach to robot design would not allow soft robots to perform these actions. According to our definition, a capability does not connect to a specific field of application. On the other hand, applying a skill may finally materialize in various sectors. Human-AI interaction, medical and surgical applications, wearable and rehabilitative robotics, space, exploration, geography, and mobility are prospective markets for soft robots.

### 4.1 General sectors

#### 4.1.1 Military applications

Self-healing soft exoskeletons have the potential to provide warriors with both protection and support. Furthermore, they may enhance the soldier’s efficacy in transporting substantial loads and navigating challenging topography (Rus and Tolley, 2015). Using self-healing soft search and rescue robots is a viable solution for effectively maneuvering through challenging areas and executing intricate operations. Additionally, it is possible to engineer these devices with self-healing capabilities, enabling them to fix any damage incurred during a rescue operation autonomously (Hardman et al., 2022).

#### 4.1.2 Walking and umping robots

Modeling these robots after hind-legged animals’ skeletons may improve their walking speed and stability performance by incorporating biological factors into the design process. Bioinspired autonomous-legged robots can save nuclear reactors and earthquakes. Cockroach-inspired FLEX1 is the first polymer-actuator-powered legged robot (Eckerle et al., 2001). Robots with six legs walk like bugs. Springs and bowtie-shaped differential electromagnetic actuators give each leg two degrees of freedom. Every joint spring balances the actuator. Batteries, power converters, and control boards define robots. Inhomogeneity and deterioration in the actuator induce sluggish mobility, and the supporting frame reduces the actuator’s power density (Youn et al., 2020). The proposed concept involves the development of a self-healing soft robotic walker constructed using a polymer matrix. This matrix has microcapsules implanted inside it, carrying a healing agent. Upon sustaining injury, these microcapsules release the healing agent, facilitating the repair process of the robot. This feature enables the robot to perform self-repair and maintain its locomotion capabilities autonomously (Terryn et al., 2021). Jumping is a common way to traverse rough terrain in the wild. Soft bodies may let jumping robots absorb the impact of landing without needing sophisticated landing abilities, allowing researchers to investigate quick manufacturing customization. Soft bodies are made possible thanks to rapid manufacturing processes (Tolley et al., 2014). The combustion-powered robot features a multi-material 3D-printed stiffness gradient body. The robot can now jump. Explosive chemical reactions increased the speed and power of the Pneumatic Network, allowing soft robots to jump (Minori et al., 2022).

#### 4.1.3 Peristaltic locomotion

Peristaltic locomotion in self-healing soft robotics involves controlled deformation and relaxation of supple and malleable robot parts. The creature moves like a worm or the intestines by contracting and expanding these segments. Peristaltic locomotion in self-healing soft robots allows them to negotiate varied terrains and overcome barriers, giving them an edge. The robot’s body is flexible and conforms to the terrain. It reduces the possibility of barriers or injury. Activating the material’s self-healing properties allows it to mend minor damage like rips or punctures, ensuring the robot’s continued functioning (Laschi et al., 2016). Invertebrates with few or no limbs, such as Oligochaeta, frequently use peristalsis. Oligochaeta must contort their bodies to perform the critical operations of locomotion (Seok et al., 2010). The actuator comprises a polymer matrix that incorporates microcapsules housing a healing ingredient. In the event of actuator damage, the microcapsules rupture, releasing the healing agent that facilitates the repair process. The actuator underwent testing under diverse settings, demonstrating its ability to repair itself upon injury autonomously (Roels et al., 2022). Peristalsis may provide muscular mobility in confined regions without complex limb structures. These include constrained space applications.

#### 4.1.4 Climbing

Self-healing soft robots have been developed to adapt to the specific characteristics of the surfaces they traverse. The flexible and deformable characteristics of these entities enable them to adapt to imperfections and roughness present in the climbing substrate. The self-healing gripper can securely grasp objects and traverse diverse surfaces. The gripper can be constructed using a material that can self-heal after injury. This enhancement enhances the durability and reliability of the gripper, enabling it to traverse surfaces that would otherwise cause harm to conventional robotic systems (Laschi et al., 2016). Bendable robots are less likely to harm themselves or their environment, making these technologies valuable. The lack of soft climbing robots is puzzling, as they can tolerate falls. As “universal,” they can travel any route (Schiller et al., 2019). Wall-climbing robots use soft matter and biomimetic technology. Stickybot employs animal-inspired hierarchical structures, tangential contact force control, and guided adhesion for stickiness. The pattern replicates animal adhesion. To distribute weight and simplify attachment and removal, the robot’s feet and other pieces include arrays of tiny, angled polymer stalks. Bioinspired force control methods, innovative manufacturing techniques, and soft-matter technologies provide robot-compliant structures (Li et al., 2016).

#### 4.1.5 Elongation and shortening

An “elongation” is a self-repairing soft robot’s ability to stretch or extend its structure or pieces. Soft robots can reach, grip, and manipulate objects in their surroundings because of the trait above. Pneumatic actuators, shape memory alloys, and flexible materials that bend and revert may allow elongation. In contrast, shortening is the robot’s capacity to shrink. Retracting limbs, folding or compacting robots for travel or storage, and adapting to tight spaces need shortening mechanisms. Shortening mechanisms may let self-healing soft robots repair or replace broken sections by retracting and extending new components (Cheng et al., 2023).

Flexible cephalopods like octopuses make good soft robotics. The flexible octopus arm can educate robot arms. Mollusk muscular hydrostat muscle architecture affected longitudinal and transverse geometry. The robot OCTOPUS can accomplish the tasks above using SMA springs in braided conical sheaths. Swimming limbs The problem of underwater legged movement, which might enable benthic zone investigation, remains despite robotics research. Robots have extensively tested it for negotiating tricky terrain (Guanjun et al., 2017). Models for the underwater legged movement that use proper compliance of the legs have been created, and these models have been based on the octopus as their inspiration. The OCTOPUS and the PoseiDRONE, which have legs made of silicone, can move about in many different ways (Laschi et al., 2012).

#### 4.1.6 Stretchability

Stretchability is essential in self-healing soft robotics because it enables the robot to bend and adapt to its surroundings, increasing its versatility and toughness. The capacity of a pliable robotic system to undergo elongation and distortion without enduring lasting harm is of utmost importance for a wide range of practical implementations, including but not limited to soft grippers, wearable apparatuses, and robots inspired by biological organisms. Stretchable self-healing materials, characterized by polymers with dynamic covalent bonds or reversible chemical interactions, facilitate the robot’s ability to restore its structural integrity after mechanical damage incurred by stretching or deformation (Agarwal et al., 2016). Imitating mollusks’ flexibility, elasticity, and conformability, a hyperelastic light-emitting capacitor (HLEC) created a thin rubber sheet with light emission and a touch sensor. The invention permitted this. HLECs with alternating ZnS elastomer and transparent hydrogel electrodes may generate flexible rubber sheets with controlled brightness and capacitance. A three-chambered, worm-like robot wears these skins. The worm became flexible, multicolored, and sensitive to its surroundings and body (Lu and Kim, 2014).

#### 4.1.7 Adaptable grasping

In self-healing soft robotics, flexible grasping means they can dynamically alter their grip to changing environmental circumstances or object attributes. Self-healing soft robotic grippers can snugly hold various objects, unlike rigid grippers, which need precise programming and may struggle with unusual forms or delicate materials. The gripper can reconfigure and rebuild its hold due to self-healing materials’ capacity to recover from harm. Adaptable grasping in self-healing soft robotics improves robustness and resilience in numerous applications. Adaptable grabbing may expedite pick-and-place, packing, and quality control in manufacturing and logistics, where products arrive in all forms and sizes. Although the item may move or distort during gripping, the soft gripper can adjust to its shape and hold securely (Zhuo et al., 2020). Soft-bodied robots and other gadgets increase technology because of their adaptability. Even though robotics has extensively studied grasping and manufacturing, prosthetics and other industries have excellent grippers, and soft materials may be used as an interface for grasping activities (Zhou et al., 2017). Soft materials can conform to any shape, and making the robot flexible increases its safety. This approach has produced several soft grippers utilizing various technology. Tendon-driven techniques work with stiff connections and adaptive grasping because they focus on the tendon (Dong et al., 2018). Hard and soft elements allow wearable technology to add functionalities. A tendon-driven solution helps disabled hands move, and this approach is used in the wire-driven Exo-Glove. Flexible fluidic actuation is an exciting example of how technology may be utilized in rehabilitation and assistance. The glove is entirely flexible while worn.

Elastomeric twisting actuators connected with hand fingers allow motion (Polygerinos et al., 2015). A soft, tunable gelatin robot can be used for grasping, transporting, and delivering small particles, liquids, and even living organisms. The robot is made of a gelatin material that can be tuned to different stiffnesses. The robot is soft, supple, gripping, and rigid when transporting and delivering. Self-healing robots can fix themselves. The robot grasps, transports, and delivers using a soft, adjustable gelatin substance and an insect-like claw. The soft, adjustable gelatin makes the robot soft and compliant for gripping and rigid for transporting and delivering. The robot can precisely grip and release things with its insect-like claw (Yang et al., 2022).

#### 4.1.8 Pulsed-jet swimming

In the field of self-healing soft robotics, the ability to flexibly grasp objects allows these robotic systems to adapt their grip in response to changing environmental conditions or the characteristics of the objects being manipulated. Self-healing soft robotic grippers can securely grasp diverse items, unlike rigid grippers that need meticulous programming and may encounter difficulties when handling atypical shapes or fragile materials. The gripper can rearrange and reconstruct its grasp due to the inherent capability of self-healing materials to recuperate from damage. The enhancement of robustness and resilience in many applications is achieved by using adaptable grasping in self-healing soft robots. The use of adaptable grasping techniques can enhance the efficiency of pick-and-place operations, packaging procedures, and quality control processes within the domains of manufacturing and logistics. Despite the object’s potential for movement or distortion while grasping, the soft gripper can adapt to its form and maintain a solid hold (Laschi et al., 2016). Cephalopods’ soft bodies and deformability inspired pulsed-jet swimming. A funnel propels water, and to modify thrust varies the contractions that eject water. Combining pulsed-jet propulsion fluidodynamics with a soft body deformation framework enhanced the PoseiDRONE’s driving body. This has resulted in the robot swimming more effectively (Renda et al., 2015).

### 4.2 Medical sectors

#### 4.2.1 Surgery

Minimally invasive surgery involves using self-healing soft robots to conduct surgical procedures via tiny incisions. These robots can be engineered with flexibility and compliance, rendering them highly suitable for performing tasks inside the human body. Additionally, it is possible to engineer these devices with self-healing capabilities, enabling them to fix any damage incurred during surgical procedures autonomously. This intervention could decrease the likelihood of infection and associated consequences (Runciman et al., 2019). The last 30 years have seen a movement beyond open surgery to invasive, less traumatic, and faster methods, improving patient outcomes and minimizing postoperative pain and scarring. Abdominal surgeries benefit most from MIS or minimally invasive surgery (Richardson et al., 2000). MIS inserts long, stiff devices into the insufflated abdomen. The rigid Lapar endoscope and 10–15-mm incisions accomplish this. Particular technologies enable MIS to reach organs through their existing channels. A flexible endoscope with many tiny devices creates an organ wall hole in the mouth, vagina, or anus. An external surgeon may operate on the organ. Endoscopy exploits the patient’s natural opening. Minimally invasive surgery may harm vital organs. Complex or semi-rigid devices with restricted motion need five abdominal incisions. NOTES and single-port surgery may be difficult due to reach or tool obstruction. Stiff instruments, mechanical linkage, and cable-actuated solutions improve surgeon accuracy (Vyas et al., 2011). Endoscopy and catheter-like operations need less precise but more flexible equipment to cross intricate pathways and reach distant organs. Surgical endoscopy makes these approaches as precise and efficient as MIS, only in life-threatening situations. The soft robotic device must have adjustable stiffness, organ compliance, and safety even if it does not induce long-term immunological reactions (Garcia et al., 2021).

#### 4.2.2 Drug delivery

Endoscopic and surgical equipment cannot reach the most inaccessible brain, liver, and pancreatic sections, nor can surgically implanted devices replenish medicine. Wire-based surgical instruments are stiff and cannot reach remote areas or offer exact dosages. Soft robots break down and release pharmaceuticals using hydrogels and other biocompatible and biodegradable materials (Kim et al., 2022). The systemic injection is worthless, but physicians use it to regulate release. Medicine may spread through electromagnetic waves, ultrasound, and warmth. Micellar liquids, nanoparticles, and thin films transport drugs. Tablet and capsule issues spurred thin-film technologies in the 1970s. Depending on composition, thin films may be a few nanometers to several hundred micrometers thick. Flexible, non-covalently sticky, molecule-permeable, rich in surface area and aspect ratio, thin films may contain many medications. Programmable passive polymers with magnetic or optical nanofillers improve thin films and material reactions. Passive drug administration may cause tissue reactions at high doses. Controlled release passive. Environmental signals for medicine timing and placement may prolong chronic illness therapy (Vannozzi et al., 2018). Using self-healing soft robotics is a promising avenue for targeted medicine delivery inside the human body. These robots can be engineered with biocompatibility and biodegradability attributes, ensuring their non-hazardous nature to the human body and eventual decomposition over time (Talebian et al., 2019). Additionally, it is possible to engineer these systems with self-healing capabilities, enabling them to repair any damage incurred during medication distribution autonomously. This approach could enhance the efficacy of medication administration and reduce the likelihood of adverse reactions.

#### 4.2.3 Rehabilitation

Robots designed to assist the elderly might be helpful. Soft robots boost independence and collaboration. Stiff-component robots stiff components help individuals with daily jobs (Fiorini et al., 2021). Self-healing soft robots can contribute to rehabilitation after surgical procedures or physical trauma. These robots can be programmed with gentle and supportive behaviors, making them highly suitable for deployment in patients undergoing postoperative or rehabilitative processes. Additionally, it is possible to engineer these systems with self-healing capabilities, enabling them to fix any damage incurred during their operation autonomously. This intervention can expedite the healing process and decrease the likelihood of further harm (Liu et al., 2021).

#### 4.2.4 Wearable robots

Active adaptation in robotic systems may be enabled through compliance and impedance control on rigid robots or flexible soft robots. Complex interfaces and bioinspired actuators have created soft, wearable rehabilitation and support devices (Banerjee et al., 2018). Self-healing soft robots may be modified to match each user, which increases their adaptability and use. The three key attributes of the product under consideration are durability, comfort, and safety (Li et al., 2020). User-adjustable portions of these systems remain closed. Soft mechatronics operates well. Lower limb primary assistive devices include stiff interfaces and patient-attached rotating joints. Soft actuators move joints. Flexible, lightweight actuators that exploit human joints may alleviate these concerns. Human touch heats SMA actuators. Therefore, they need protection. New devices and interfaces boost system performance. Pneumatic actuators enable ankle-worn robots. Wearable sensors monitor deformation and force. Chamber length affects cross-section, which affects sensor longitudinal electrical resistance—studying soft actuators on stiff linkage systems. These safe, lightweight devices use pneumatic artificial muscles for regulated compliance. Gloves touch joints. Patients may flex their fingers with torque for rehabilitation or assisted work (Pan et al., 2022).

### 4.3 Human body mimicking robots

#### 4.3.1 Prostheses

Soft robotic technology may improve prosthetic limb usage and acceptance for amputees. Soft robotics improved robotic manipulation, and the biggest problem is spreading compliance through soft mechatronic technology. Soft robotic technology has produced several artificial hands, but few are prosthetics (Xiloyannis et al., 2021). Soft robots’ delicate yet effective grabbing might aid non-biomedical fields, including food and underwater exploration. Portable, controlled devices enhance soft robotic prostheses. Rehabilitation and assistive tech follow these standards. Similar approaches have been proposed for prosthetics. Researchers are developing more comfortable prostheses. Soft robotic technology may make hard-material prosthetic limbs more flexible to the wearer’s evolving body. Poor socket adjustment makes lower-limb prostheses hard to use. Too firm or soft sockets might create stump sores or moisture issues, while too soft can cause instability and uneven walking (Cruz et al., 2020). Using self-healing soft robotics is a promising avenue for enhancing the durability and reliability of prostheses and orthotics. These gadgets have the potential to be constructed using self-healing materials, which possess the ability to fix any damage incurred autonomously. This practice can enhance the equipment’s longevity and decrease the need for maintenance (Khatib et al., 2021).

#### 4.3.2 Artificial organ

Artificial organs are medical devices that replace or improve organ function. Their design may use metals, plastics, and polymers. Self-healing materials fix prosthetic organs. Use or modify shape memory polymers for self-healing. Many ways exist for self-healing artificial organs. Self-repairing biomaterials may enlarge the artificial organ. Deformed shape memory polymer prosthetic heart valves may reform. Therapeutic microcapsules can self-heal artificial organs. Microcapsules rupture after artificial organ damage, releasing a healing chemical that restores. Self-healing artificial organs requires reversible connections. Artificial organ damage breaks reversible connections. Linkage restoration may fix the damage. Self-healing artificial organs might improve medicine’s durability and dependability. They may also make long-lasting prosthetic organs (Jain et al., 2022). Many researchers have developed artificial cardiac systems. Most of it has focused on mechanical needs rather than soft robotic technologies. (achievable pressure, frequency, and sometimes working cycles). Four SMA plates and bias springs allow manual or shapeshift-induced urethral opening. It is warm and sluggish (Gerosa et al., 2014).

#### 4.3.3 Body-part simulators

Soft robotics uses malleable materials, contractile actuators, and bending sensors to create body-part simulators. Realistic human body models have been constructed to instruct specialists in high-fidelity healthcare simulation situations and study human physiology (Li et al., 2022). Simulation software can develop realistic stimuli-response simulators. These simulations may instruct surgeons. Users may use virtual tools by reacting to force, torque, and displacement. However, tactile feedback is rare. Simulations should use accurate human body models. In an EVE simulator, actuating mechanisms simulate the heart and arteries. The simulator may use soft sensors to simulate endoscopic and intubation operator touch and force. Japanese Bionic Humanoid proposes a human prototype with sensors and actuators to replace animal experiments. Bionic-EyE simulates chemically crosslinked PVA hydrogel eye surgery. Materials for human physiology models must match tissue mechanical characteristics. The commercial silicone chemistry can vibrate three-layer voice cords like human ones. When developed, this simulator duplicated ordinary functionality. Expect polyps and hypertonic vocal cords. Bioinspired swallowing robots may study dysphagia. Soft robots are silicone-bodied. Several inflatable chambers and a flexible sensor grid simulate swallowing (Chen et al., 2013). Centralized pattern generators indicate peristaltic waves. A soft robot that mimics tongue movement may study tongue movements and interactions. This artificial mouth mimics human tongues well but cannot match their intricacy. It is made of silicone rubber for an extended layer and PDMS for a network of inner chambers. Due to tension on its inner and outer layers, the mechanical tongue deflects (Martinez et al., 2014).

## 5 Implementation challenges and potential approaches

Variable stiffness and soft robots are evolving. To apply a physical principle that underlies observable macroscopic behavior to the development of a new actuation technology, one must first a) represent the phenomenon, b) investigate its material properties, and c) develop a comprehensive theory that accounts for its structural implications. Soft bio-inspired robots have materials, design, and system integration issues. Even with comprehending soft bio-inspired robot technology, unanticipated obstacles may develop. Self-healing robots are still developing, requiring new applications despite extensive research. Robots are new to the real world and have limited uses.

### 5.1 Soft motion

SHSME’s hierarchical polymer network features many reversible connections. Robots can endure physical stress, although their softness makes cuts and piercings more frequent. Alpha lipoic acid causes SHSME hierarchical dynamic linear chains and crosslinkers. (ALA). SHSME is suited for soft robotic applications that necessitate speedy damage recovery. Since it is thermoformable, it is auto-repairable (Collins et al., 2005; Pfeifer et al., 2007).

### 5.2 Environmental compatibility

Traditional robotics seeks longer-lived robots, and retired robots will hurt the environment. More soft robots need adaptation and post-life effects. This problem requires biodegradability. Eco-friendly biodegradable robots are required. Recently, pneumatically and electrically driven biodegradable biopolymer soft actuators functioned. The breakdown of various plastics reminds us that safe deterioration is not the same as breakdown. In many cases, even shattered plastic may affect the ecosystem. Environmental soft robots must demonstrate that their short-term and long-term environmental impacts are neutral or favorable (Chambers et al., 2014; Wang et al., 2016;).

### 5.3 Design automation tools

Soft systems are intricate and cannot be guessed. Design automation is required. Due to regular exposure to mechanical equipment, many professionals base their design decisions on their intrinsic grasp of stiff object kinematics and dynamics. These technologies will improve intuition and may require design automation tools to explore the design space and achieve higher-level goals. Design automation technologies demand new mathematical models and accurate, fast-processing simulators (Verl et al., 2015).

### 5.4 Actuation methods

Soft actuators must be more solid and practical despite the widespread adoption of soft materials for construction and sensation. Electroactive polymers need high voltages, while low-voltage IPMCs are weak, slow, and inefficient. Shape-memory wires need much electricity, while pneumatic actuation requires an extensive pressure infrastructure. These actuation issues make untethered soft robots problematic (Reynolds and Holwell, 2010; Shintake et al., 2018).

### 5.5 Control authority

Soft-material robot control requires precision and authority, and feedback control may fine-tune speeds and locations via estimate and correction. Soft materials lower impedance, but lag times, deformations, and vibrations complicate control. Additional sensing and modeling may be needed to identify answers, but new control techniques and design paradigms that cope with the larger design space will be needed (Jiang et al., 2020).

### 5.6 Simulating soft robots

Extreme deformations bend, flex, and collapse a material, rendering linear approaches impossible. The kinematic modeling approach involves nonlinear relaxation techniques, which include dividing the structure into discrete elements such as springs, beams, and masses (Grazioso et al., 2020). Soft materials have variable flexibility and nonlinear behavior, making modeling difficult and time-consuming. Modeling nonlinear effects requires complex computations. Even a perfect simulation requires experimentally calibrating friction, nonlinear elastic characteristics, damping coefficients, and material surfaces to match simulation results to real-world observations (Bark, 2019). Simulate each component’s local dynamics and kinematics. Technically correct simulators cannot guarantee accurate predictions. Experimental data must calibrate material properties for a natural match. Depending on model component complexity and element type, these properties may need significant physical testing to calibrate. Open-source light matter physics framework Voxelyze and GUI-based VoxCAD (Horibe et al., 2022). It is proposed to use a combination of modeling, simulation, and biological material methodologies. Novel modeling and simulation techniques for soft bodies and materials in robotics are in demand. Existing models and simulations must depict soft robots’ complicated actions. Soft materials are essential in robotics because they need continuous models and discrete mechanical joint simulation. Soft robots have centralized and dispersed controls.

Some Potential Approaches can be Design automation, Proper representation, and Proper representation. Self-healing and damage-resistant soft robots are promising research subjects. Today, self-healing soft robots encounter challenges yet have immense potential. Multifunctional materials that bridge functional partitions are driving soft robotics progress. Therefore, self-healing materials’ potential to destroy functional barriers must be stressed. Developing this topic requires authentic materials and modeling and simulation tools. Complex application circumstances and growing labor costs have led firms to automate manufacturing processes, requiring modeling, simulation, and material interaction tools (Iida and Laschi, 2011). Simulate complicated processes. Soft tissue differences and their effects on complex sensory-motor systems may complicate the body. Multiple studies have shown that this strategy improves the body and governs (Bongard et al., 2006). Modeling plastic morphologies and material quality is a critical soft robotics problem. Plastic morphological change models may mimic optimization across several timescales.

Designing with soft materials requires design automation. Design automation technologies can quickly explore the design space, generate new ideas, and improve present designs since individuals cannot instinctively predict soft materials’ behavior and interaction. Design automation involves accurate physical simulation and analysis (Hu et al., 2019). Physical simulations can optimize different geometries and multi-material combinations to achieve design goals. Mixing hard and soft materials may provide a lightweight structure supporting weight. Optimizing a complicated material block might add, remove, and swap components to optimize efficiency.

Design automation systems define potential designs and optimize options using algorithm—encoded robot characteristics. Solution 3D voxels may indicate material choices via direct encoding. Straightforward gradient descent might enhance the material distribution from a random voxel assignment until it can no longer improve (Lee et al., 2020a). Soft robot simulation and design representation may inspire evolution. Compositional pattern-creating networks (CPPNs) encode spatial functions and design robot morphologies by specifying the material type in coordinates (Lipson, 2014).

## 6 Opportunities and future Scopes OF SH soft robotics

Soft robotics is developing technically and systematically. Since its founding, researchers have studied unique materials, morphologies, functions, self-organizations, self-stability, and self-assembly. Modeling is complex, with several degrees of freedom. Several simulation methods approximate soft robotic system behavior with non-linear kinematics and considerable material deformation. Despite advances, models are complex, and approximations allow significant analytical errors.

Soft robotics is modern. For this challenging application, soft robotic manipulators require active chemicals and strain, tension, and velocity. Materials science is essential to large-scale actuator development but is not the only factor (Sun et al., 2016). Soft robotics may enable the creation of high-performance, human-safe robots. Due to design, modeling, and operating challenges, such robots have immense potential but are difficult to realize. Soft robotics, which uses specifically designed components, gadgets, and gear to bridge the gap between people and robots, has advanced (Coyle et al., 2018). These emerging technologies may improve human-robot and robot-environment interactions. Biomechanically compatible soft bio-inspired robots may benefit healthcare, mobility, and disaster relief.

A tiny subset of elastomers and compressed air or vacuum has powered most soft robotics research. Alternatives, particularly energy-based ones, are popular (Golchin et al., 2021). Amorphous shapes and flexible motion allow greater freedom of movement and form than rigid mechanics. Robots that move and manipulate in novel ways fall into this category. Additive printing technology, which can handle soft and graded materials, is one example of a new manufacturing technique that dramatically increases design flexibility (Scalet, 2020). Hybrids with chemical fuels and electrical actuators/controllers should benefit from fuel cells compatible with soft materials, but this has yet to be studied. Soft supercapacitors are also being investigated (Yang et al., 2020). Other actions, such as strain release in tightly coiled, pre-stressed fibers, may benefit specific applications.

Chemists can engage in synthesizing novel chemicals, materials, and industrial techniques to propel the field of chemistry forward. Soft robotics in the context of human healthcare necessitates specific characteristics, including biocompatibility, safety, *in vivo* usability, non-contact detection and communication capabilities, and predictive capabilities (Chowdhary et al., 2019). Conventional robots excel at one task but require reintegration and retraining when their roles change. Retraining a machine for a new function takes hundreds of hours and is costly. Food, consumer goods, and other industrial sectors created soft robots to address this problem.

Soft robots used in industry are called collaborative robots. Industrial robots have traditionally followed a linear production process (Ansari et al., 2015). Soft robotics is a good alternative for human hands for mild handling tasks. Soft robotic hands mimic human hands to avoid mishaps while assembling heavy or pointy things. These robots can enhance output and safety. Industrial and consumer robots are stiff, and robotics is popular in toys. Carnegie Mellon University’s Morphing Matter Lab has found promising robot applications in this sector (Chin et al., 2020). Knitting machines produce strong motor tendons, and their product embraces the stomach when touched. Robots will produce clothes and accessories using this idea.

Recent advances in soft robotics have improved robotic systems’ capacity to reproduce and adapt to actual conditions. Soft computers let us apply biological notions. Incompatibility with other machines prevents the widespread adoption of human-interfacing robots, which might benefit the home and business. The product’s safety, functionality, and impression depend on its components’ compatibility. Soft robots may appeal to non-robotics experts. Medical and prosthetic robots might utilize soft matter (Lipson, 2014).

Innovative actuators enable soft robots with unusual movement, strength, and reliability. Conflicting and poorly defined design goals impede the design process. In the same way that biological continuum manipulators use complex and discrete components and operations to simplify their interactions with their environments, these robots would perform better in most tasks by using a well-balanced combination of continuous, soft, discrete, and complex elements (Ashuri et al., 2020).

Soft robots need advanced controls, accurate modeling, and efficient production. Soft robot success depends on sensor and actuator development, and soft material research will provide affordable, functional alternatives. After tackling these issues, soft robots may expand faster (Trivedi et al., 2008).

Soft robotics uses pneumatic energy. Pneumatic systems might become the norm, increasing system capabilities. Combustion-based alternative fuels may work. Methane and butane have been examined, but their quick and high-pressure reactions exceed current control methods. Explosive gases may only be utilized for power when alternative energy sources are researched or the system level is upgraded (Wehner et al., 2014).

Stretchable electronics are prototypes of wearable electronics. Today, humanoid robots and E-skins are socializing. A pressure-sensitive rubber grid of organic field-effect transistors forms the electronic skin. (E-skin). Rehabilitation gloves might be mass-produced if a new method can be found. Flexible electronics will improve physical therapy and other human-compliant applications (Lu and Kim, 2014).

Grid size precision is a problem for tactile sensors and flexible electronics. Increasing the grid size resolution is necessary for the future so that they can cover more expansive and complicated regions. Better sensors and electrical control are also required to support the smaller grid sizes. If the grid were smaller, the electronics and sensors could be used in more places (Tajima et al., 2002). Studies have shown that biomedical soft robots still need to be at a level of sophistication where they may be safely used in clinical settings. Problems, including high upfront costs, poor usability, and a disconnect between technological progress and actual patient demand, contribute to this barrier (Song, 2016).

Soft robotics tries to design a system that can self-modify its behavior. Adhesion research is enabling larger-scale autonomous morphing applications. Medicine, education, micro-air vehicles, and deformable sensors may enhance this sector (Mazzolai et al., 2022).

Soft robotics may enable the creation of eco-friendly artificial systems. Future soft bioinspired robots will use sustainable energy, be recyclable or biodegradable, and adapt and evolve. These soft green robots, a new generation of eco-friendly technology, will imitate nature by regenerating.

## 7 Conclusion

This paper studies soft robotics using self-healing material. Soft robots have malleable bodies and electronics. Soft robotics builds soft robots. Fully soft-bodied robots might do marvels. They can fit where rigid items cannot due to their flexibility. It is best to implant or interact with a soft robot. Soft robots that can heal themselves are the only way to preserve the industry’s fragility. Self-repairing polymers allow soft robot repair and reuse.

Soft robot shape and motion sensing and control take careful attention. Actual soft robots have finite sensors and actuators, but theoretically, they have unbounded freedom. Soft robots’ degrees of freedom are sometimes impossible to see or control. Modeling soft robot large-deflection dynamics is expensive for real-time control. Model order reduction and efficient solution approaches may provide fast, accurate dynamic control models. Inverse dynamics models may help soft robot feedforward control. Innovative additively made or molded materials are essential to soft robotics. For energy-efficient adaptive and integrative topologies with essential properties, robotists need materials. Additive manufacturing should perform like predictable soft materials.

Early self-healing polymers employed encapsulation, reversible chemistry, and finally exploited vascular systems. Modern self-healing systems merge. Soft robotics is a promising SH polymer application despite its severe limitations. Developing regulated stimulation equipment and condition monitoring sensors is necessary due to the human body’s flexibility. A robotic system sets the stage for these systems. SH technology is considered for robots, cars, and other machines.

This review links soft robotics with self-healing materials. According to preliminary literature, fatigue, delamination, overloading, tendon wounds, and sharp objects may destroy soft robots. Because of this, the ability to heal has become more critical for repairing these robots and reducing future environmental and maintenance costs. This innovative combination requires more research to align material capabilities with the soft robotics industry’s self-healing soft robot systems demands. It is investigating the multidisciplinary field’s aims and limits.
